# Extracting Additional Influences From Physician Profiles With Topic Modeling: Impact on Ratings and Page Views in Online Healthcare Communities

**DOI:** 10.3389/fpsyg.2022.830841

**Published:** 2022-04-01

**Authors:** Xiaoling Wei, Yuan-Teng Hsu

**Affiliations:** ^1^College of International Business, Zhejiang Yuexiu University, Zhejiang, China; ^2^Research Center of Finance, Shanghai Business School, Shanghai, China

**Keywords:** online healthcare community, text mining, topic modeling, ratings, page views

## Abstract

How physicians can get better ratings and more page views in online healthcare communities is an important issue. Based on 38,457 physicians' profiles from a popular online healthcare community in China, we used Latent Dirichlet Allocation model, which is a common topic model, to analyze the non-English text to obtain more doctor's latent characteristics. We found five of the most frequently mentioned topics. In addition to the first topic (doctor's academic rank and practice name), “research ability,” “foreign experience,” “committee position,” and “clinical experience” were included as unstructured descriptions in the doctor's profile. Inferences about physician ratings and page views could be improved if these themes were set as characteristics of physicians. Specifically, in our findings, Physicians' mentions of their “research ability” and “foreign experience” had a significant positive impact on physician ratings. Surprisingly, physicians mentioning more “clinical experience” had a significant negative impact on physician ratings. Moreover, while descriptions about “foreign experience” and “committee position” had a significant positive impact on page views, physician mentions of “research ability” had a significant negative impact on page views. These results provide new insights into the ways in which online healthcare community managers or physicians create their personal online profiles.

## Introduction

Online healthcare communities (OHCs) can help patients get more medical information, find the right hospital or clinic department, and choose the right doctor based on their profile and relevant online reviews. In addition, many OHCs provide medical consultation services, allowing patients to receive medical assistance by phone or video. Thus, OHCs can help reduce stress across the healthcare system and improve rural-urban health disparities (Tu et al., 2015; Goh et al., 2016), which have important functions in the wake of the COVID-19 epidemic. However, in the development of successful OHCs, physicians play a crucial role due to the inherent expertise of medical knowledge (Guo S. et al., 2017; Wang et al., 2017). For OHC managers who are responsible for setting policies, designing user interfaces, and managing members, further parsing various characteristics or information about physicians is a key challenge for OHC development. Such features are useful to identify highly qualified physicians or identify ways to make physicians more attractive to users.

In other words, we would like to know what characteristics doctors possess to be most influential in OHC. Specifically, this study measures physician influence in OHC from two perspectives. First, if physicians' electronic word of mouth (eWOM) is better, it will help OHC in the long run. Physician ratings are the most widely used proxy for the value of eWOM^1^ (Liu, 2006; Dellarocas et al., 2007). Previous studies usually show a positive correlation between average ratings and sales of different products (You et al., 2015; Rosario et al., 2016). Secondly, the most important question is whether physicians can attract more people to participate in this community and thus increase the number of page views on the website. From the perspective of OHC managers, more website visitors may be one of the most important indicators of OHC development (Demers and Lev, 2001; Dewan et al., 2002; Luo et al., 2013).

Based on the above discussions, which characteristics of physicians can be associated with better ratings and attract more users to participate in OHC is a topic worth exploring. In past studies, the available information about physician characteristics mainly come from two sources. First, most OHCs reveal the physician's name, title, education, and the hospital department to which the physician works for. Second, the user-generated data in OHCs should be noted. The ratings and views mentioned in the previous paragraph belong to such features. In addition, prior studies have used text analysis to extract certain information from reviews provided by patients as characteristics of doctors, for example, by calculating the average sentiment score of reviews. It is worth noting that OHC usually enables physicians to fill in a personal profile, which provides additional explanation and context to increase the diagnostic nature of the information (Mudambi and Schuff, 2010). We believe that personal profiles may contain a wealth of information about a physician's academic, foreign, administrative, and clinical experience, yet limited research has been conducted in the past literature to focus on this component. This study intends to extract additional features from physician profiles using text mining, as well as analyze whether these features can be used to explain physician ratings and page views in OHC.

We collected 38,457 physician profiles from the Haodf website (http://www.haodf.com/)^2^, which is one of the most popular OHCs in China, and then extract physician characteristics from this text data. Intuitively, if a doctor's profile expresses a latent characteristic, then some specific words will appear more frequently. The topic model in text mining is a statistical method used to discover abstract topics from a large amount of text. This study adopts Latent Dirichlet Allocation (LDA) to perform the analysis (Blei et al., 2003), which is one of the most common topic models. After extracting the new characteristics of doctors by LDA, we use regression models to verify whether these features have an impact on the ratings and page views of doctors.

## Literature Reviews

### Online Healthcare Community Development Status

The online healthcare community (OHC) has become a new venue for online physician-patient interaction (Goh et al., 2016). As OHC users, patients can search for health-related information, exchange experiences, benefit from social support, and conduct online consultations with professional physicians (Johnston et al., 2013; Atanasova et al., 2018). As an Internet-based platform, the OHC connects people with information relevant to their health-related interests or problems; therefore, OHC sites are important venues for people to connect with others who have similar health conditions. In general, OHC sites can be divided into two types. The first is OHC sites, used primarily in peer support groups and often referred to as online support group sites (Barak et al., 2008). There are a variety of health-related online support groups, such as those for people living with HIV/AIDS (Mo and Coulson, 2010), breast cancer (Høybye et al., 2005; Radin, 2006), food allergy (Coulson and Knibb, 2007), and so on. The second type of site, usually associated with the term OHC, is comprised of online sites used by patients and health professional moderators, typically health care professionals or physicians. In the latter type of OHC, health professional moderators provide reliable health-related information and professional health consultations (Johnston et al., 2013; Zhao et al., 2013; Petrovčič and Petrič, 2014). The most popular OHC sites in China (i.e., Haodf, Chunyuyisheng, and WeDoctor) usually offer professional health-related information and physician-patient interaction.

The importance of the OHC is growing and changing users' conceptions of face-to-face medical encounters, broadening professional-patient interaction channels (Guo S. et al., 2017). There are three primary groups of OHC stakeholders: purveyors, patients, and physicians. We define purveyors as planners or designers of the platform. Due to the friendly online interaction environment provided by the purveyors, these platforms attract physicians and patients to participate actively in them (Blut et al., 2015). Users and patients of OHC can not only interact with their personal physicians, but also consult with other health professionals (e.g., specialty physicians), they are able to receive increased amounts of information (Atanasova et al., 2018). Physicians can share medical or healthcare knowledge with patients through the OHC, and the benefits of the OHC for participating physicians include social returns and economic returns (Guo S. et al., 2017). Previous research on the OHC has primarily been conducted from the users' (or patients') perspectives (Vennik et al., 2014; Yang et al., 2015) and from physicians' perspectives (Guo S. et al., 2017; Guo et al., 2018), while limited studies explore OHCs from purveyors' perspectives. This study which analyzes unstructured data to extract information can provide purveyors with more ideas about website design management and advertising strategies.

### Applications of Text Mining

The variety of text analysis tools and approaches for managing and analyzing unstructured data is growing rapidly (Balducci and Marinova, 2018). These options provide exciting new ways to gain insights into some of the problems and questions that have been identified as new areas for research. Text mining is the most fundamental approach which involves the extracting of meaningful information from text. Traditionally, text-based analysis of user-generated content (UGC) has drawn much attention in the recent marketing literature. Most previous studies using textual consumer reviews have involved various goals in the area of marketing research, such as eliciting product attributes and consumers' preferences by mining consumer reviews (Decker and Trusov, 2010; Archak et al., 2011; Lee and Bradlow, 2011), predicting the impact of consumer reviews on consumers' purchase decisions using the valence of sentences (Berger et al., 2010), predicting the product sales and market performance of a product based on review content and sentiment (Dellarocas et al., 2007; Ghose et al., 2012; Tirunillai and Tellis, 2012; Goes et al., 2014), and analyzing the conversion rates resulting from changes in affective content and linguistic style of online reviews (Ludwig et al., 2013). In addition, the topic model involves the use of well-known and important modern machine learning technology that has been widely used in text mining, latent data discovery, and the finding of relationships among data and text documents.

There are various methods for topic modeling; Latent Dirichlet Allocation (LDA; Blei et al., 2003) is one of the most popular methods in this field and has been widely used in various marketing applications (Tirunillai and Tellis, 2014; Büschken and Allenby, 2016; Jacobs et al., 2016; Trusov et al., 2016; Guo Y. et al., 2017; Puranam et al., 2017). For example, Puranam et al. (2017) analyzed the effect of calorie posting regulations based on an LDA with informative priors. Trusov et al. (2016) used the LDA to trace online surfing behavior, allowing online businesses to make profile predictions when limited information is available. Guo Y. et al. (2017) employ a similar approach to extract latent dimensions of customer satisfaction from rich online review data in the hospitality industry. Tirunillai and Tellis (2014) apply the LDA to consumer reviews to discover the potential dimensions of product quality, to understand the brand's position along these dimensions, and to estimate how dimensions and brand position change over time. Büschken and Allenby (2016) propose an LDA that uses the sentence structures found in reviews to improve prediction of online customer ratings. Finally, Jacobs et al. (2016) apply the LDA to the assessment of buying patterns and prediction of future purchase probabilities. In recent years, researchers have conducted text mining studies in healthcare field (Hao and Zhang, 2016; Speier et al., 2016; Shah et al., 2021a), especially during the COVID-19 pandemic, leading to a dramatic increase in the literature on LDA (Liu et al., 2020; Xue et al., 2020; Shah et al., 2021b). For example, Xue et al. (2020) analyzed the public sentiment associated with 11 selected topics identified using LDA on COVID-19 tweets. Liu et al. (2020) used a topic modeling approach to extract nine major primary themes from Chinese social media. In addition, the study by Shah et al. (2021b) conducted a number of investigations of patient online reviews in US physician rating websites to examine trends in patient attention due to COVID-19, using LDA-based topic modeling to generate topics and corresponding keywords. However, few studies have used the LDA method to analyze the profiles provided by physicians. The current research aims to fill this gap in the application of LDA.

## Research Methodology

### Data Collection

Our sample was collected from the Haodf website (http://www.haodf.com/), which is one of the most popular OHC in China. To ensure that physicians in our sample really were engaged in this site, the current study adopted data filtering rules as follows. First, only physicians with personal pages were used in our sample; this allowed us not only to reliably verify the identity of physicians but also to obtain more of their characteristics. Second, in order to avoid effects contributed by new users, all physicians in our sample joined the Haodf website before June 25, 2017. In addition, to ensure that each physician was still active on the website, the latest login time for each had to be within 1 month of the study date. Third, since this study intended to use text mining to analyze each physician's introduction, the length of the physician's introduction should be longer than 10 characters. With these restrictions, we used web crawler technology to generate the related public information on this site from May 29, 2018, to May 30, 2018. With the above filtering rules, we have a total of 38,457 physicians in our sample from a variety of different divisions. According to the classification of the Haodf website, it contains internal medicine, surgery, gynecology-obstetrics, pediatrics, orthopedics, ophthalmology, oral health, cancer, Chinese medicine, and others, a total of 10 categories.

### LDA Implementation

This study applies the LDA model to investigate what kinds of content are included in physicians' profiles in the OHC. LDA model adopts a sophisticated text-mining technique to fit a topic model (Blei et al., 2003). It regards each document as a mixture of different topics and treats each topic as a mixture of different words. We estimate these hidden parameters by implementing the variational expectation-maximization algorithm for the LDA model in R (Grün and Hornik, 2011). Regarding the use of the LDA model in our context, three points should be explained clearly. First, a physician's personal profile usually included a variety of topics, such as degree, experience, or expertise. Our goal was to discover what different topics can be found in one physician's personal profile, rather than to categorize the profile as including one specific topic. In this case, each physician's personal profile is split into several sentences by the symbol “°”, which is used as a full stop symbol in Chinese documents. Then each sentence is regarded as one individual document in the LDA model and assigns a possible topic for the profile. In this way, we investigate what kinds of topics appear in physicians' personal profiles, and the corresponding results allow us to extract additional information on the physicians' characteristics, beyond the standard information in the OHC.

Second, while a single character in Chinese generally has a complete meaning by itself, it is often necessary to combine two or more characters to obtain a meaningful token. Just as in the process of text mining in the English language, we need to remove certain stop words in Chinese (e.g., we, is, of). We also remove certain highly frequent words (e.g., hospital, doctor, China) and professional medical words (e.g., diabetes, internal medicine, cancer). This is necessary to extract meaningful topics rather than merely distinguish physicians' medical specialties. This study is implemented with the use of jiebaR, which is a well-known Chinese text segmentation tool (https://github.com/qinwf/jiebaR). Third, since the number of topics in the LDA model is assumed to be known and fixed a priori, we determine the optimal number of topics according to the perplexity (Blei et al., 2003). Specifically, the whole sample is randomly divided into two parts: 90% for the training dataset and 10% for the testing dataset. The training data are used to estimate the parameters of the LDA models, then the predictive perplexities of these trained models are calculated by using the testing dataset.

### The Empirical Model

In this study, we investigate the factors that influence the physicians' ratings (HOT) and page views (VIEW). We describe the base model as follows.


$$\begin{array}{l} HOT_{i}=\alpha_{i}+\overset{J}{\underset{j=1}{\sum }}\beta_{j}DC_{ij}+\overset{K}{\underset{k=1}{\sum }}\gamma_{k}DIV_{ik}+\varepsilon_{i}\;\left(Model\;0a\right)\\ VIEW_{i}=\alpha_{i}+\overset{J}{\underset{j=1}{\sum }}\beta_{j}DC_{ij}+\overset{K}{\underset{k=1}{\sum }}\gamma_{k}DIV_{ik}+\varepsilon_{i}\;\left(Model\;0b\right) \end{array}$$

*where i* = 1,2,…, *N* ; *j* =1,2,…*,J* ; *k* = 1,2,…*,K* ; ε_*i*_~ *iidN* (0, $\sigma_{\text{i}}^{2}$)

In the above equation for Model 0a, HOT on the left of the equal sign is the mean of overall ratings by patient reviews of physicians, the subscript *i* denotes the *i*-th physician, and there are *N* physicians in total. Next, α denotes the intercept, and β and γ are vectors of the parameters to be estimated. DC is a vector of multiple physician characteristics as a set of independent variables, and the superscript *j* indicates different items, of which there are six in total (*J* = 6) in this study: length of profile (WORD), online contribution (CONTR), tenure with Haodf (TIME), clinic title (CT), academic rank (AT), and hospital level (HL). DIV represents the physician's division, and the superscript *k* represents the different sources, of which there are ten in total (*K* = 10) in this study: internal medicine, surgery, gynecology-obstetrics, pediatrics, orthopedics, ophthalmology, oral health, cancer, Chinese medicine, and others. The distribution term ε follows the normal distribution, which makes the regression a multiple linear regression. Model 0b replaces HOT with VIEW, and the other independent variables are the same.

We determined there to be five topics through the LDA method. One of the topics is already included in the DC variables. The other four topics are research ability (RESEARCH), foreign experience (FEXP), committee position (COMM), and clinical experience (CEXP). The LDA allows us to know the keywords in each topic. When a keyword for a topic appears in a physician's profile, we label that physician as having “mentioned this topic.” For example, when the word SCI appeared in a physician's profile, we labeled that physician as having mentioned research ability in the profile and set the dummy variable RESEARCH to 1. We build these topics into four dummy variables and estimate models with the following form:


$$\begin{array}{l} HOT_{i}=\alpha_{i}+\overset{J}{\underset{j\;=\;1}{\sum }}\beta_{j}DC_{ij}+\overset{K}{\underset{k=1}{\sum }}\gamma_{k}DIV_{ik}+\lambda_{i}RESEARCH_{i}\\ +\varepsilon_{i}\left(Model\;1a\right)\\ HOT_{i}=\alpha_{i}+\overset{J}{\underset{j\;=\;1}{\sum }}\beta_{j}DC_{ij}+\overset{K}{\underset{k=1}{\sum }}\gamma_{k}DIV_{ik}+\theta_{i}FEXP_{i}\\ +\varepsilon_{i}\left(Model\;2a\right)\\ HOT_{i}=\alpha_{i}+\overset{J}{\underset{j\;=\;1}{\sum }}\beta_{j}DC_{ij}+\overset{K}{\underset{k=1}{\sum }}\gamma_{k}DIV_{ik}+\tau_{i}COMM_{i}\\ +\varepsilon_{i}\left(Model\;3a\right)\\ HOT_{i}=\alpha_{i}+\overset{J}{\underset{j\;=\;1}{\sum }}\beta_{j}DC_{ij}+\overset{K}{\underset{k=1}{\sum }}\gamma_{k}DIV_{ik}+\rho_{i}CEXP_{i}\\ +\varepsilon_{i}\left(Model\;4a\right) \end{array}$$

RESEARCH is designated as a binary dummy variable, giving 1 when the physician mentions research ability (e.g., “SCI” or “National Natural Science Foundation” or “project”) in his/her profile, and otherwise 0. FEXP is a binary dummy indicating the physician mentions foreign experience (e.g., “international” or “America” or “Japan” or “Germany”) in his/her profile. COMM is a binary dummy indicating the physician mentions committee position (e.g., “editorial board” or “standing committee” or “chairman” or “standing committee”) in his/her profile. Finally, CEXP is also a binary dummy variable set to 1 when the physician mentions clinical experience (e.g., “experience” or “many years” or “long-term”) in his/her profile, and otherwise 0. The only difference between Models 1a−4a and 1b−4b is that Models 1b−4b replace HOT with VIEW. The names, definitions and constructions of the variables and the descriptive statistics are all listed in Table 1. Table 1 also shows that the physicians received an average rating (HOT) of 3.89. The standard deviation of the rating is 0.34. The average number of views per physician's personal page is ~12, and their standard deviation is 1.83.

**Table 1:** Variable measurements and descriptive statistics.

**Code**	**Variable**	**Measurement**	**Mean**	**S.D**.
HOT	Ratings	Mean of overall ratings by patient reviews of physicians.	3.892	0.340
VIEW	Page views	Natural logarithm of the number of views for each physician's personal page on the Haodf website.	11.987	1.827
**Control variables**				
WORD	Length of profile	Natural logarithm of the number of words in the physician's personal profile.	5.218	0.992
CONTR	Online contribution	Natural logarithm of the score of the physician's contribution reported on the Haodf website.	7.632	2.415
TIME	Tenure with Haodf	Natural logarithm of the physician's tenure with the Haodf website (days), calculated by the data download date minus this physician's registration date on the website.	5.017	2.836
**Structured information**				
CT	Clinic title	Clinic title for physicians. Dummy variable, CL = 1 if the physician's position is chief physician or associate chief physician; 0 otherwise.	0.707	0.455
AT	Academic rank	Academic rank for physicians. Dummy variable, AR = 1 if the physician's academic rank is professor or associate professor; 0 otherwise.	0.516	0.500
HL	Hospital level	Dummy variable, HL = 1 if the physician is from a tertiary hospital in China; 0 otherwise.	0.783	0.412
DIV	Division	Physician's division, categorized by the Haodf website, including internal medicine, surgery, gynecology-obstetrics, pediatrics, orthopedics, ophthalmology, oral health, cancer, Chinese medicine, and others.	-	-
**Unstructured information (Latent topic)**				
RESEARCH	Research ability	Dummy variable, RESEARCH = 1 if the physician mentions research ability (e.g., “SCI” or “National Natural Science Foundation” or “project”) on his/her profile; 0 otherwise.	0.221	0.415
FEXP	Foreign experience	Dummy variable, FEXP = 1 if the physician mentions foreign experience (e.g., “international” or “America” or “Japan” or “Germany”) in his/her profile; 0 otherwise.	0.265	0.441
COMM	Committee position	Dummy variable, COMM = 1 if the physician mentions committee position (e.g., “editorial board” or “standing committee” or “chairman” or “standing committee”) in his/her profile; 0 otherwise.	0.177	0.382
CEXP	Clinical experience	Dummy variable, CEXP = 1 if the physician mentions clinical experience (e.g., “experience” or “many years” or “long-term”) in his/her profile; 0 otherwise.	0.449	0.497

*All sample were collected from the Haodf website. S.D. denotes standard deviation*.

## Results

### Topic Modeling Result

We apply the LDA to extract and label the dimensions of product introduction across all of the physicians' profiles collected in our sample. According to the predictive perplexity, we determined the number of topics to be 5 in this empirical study. The LDA identified 5 topics in which each topic showed the top-15 words by frequency. The naming of the dimensions was first carried out by one researcher and then confirmed by a second researcher. Naming was based on the identification of logical connections between the most frequently used words within the topic. Table 2 presents the results of the 5 topics generated by the model for the physicians' profiles; each topic is represented by a group of keywords. The five topics are “academic rank and clinic title,” “research ability,” “foreign experience,” “committee position,” and “clinical experience.” It is worth mentioning that in the physicians' profiles, only the first extracted topic (i.e., the physician's academic rank and clinic title) represents a structured description in his/her profile. Other topics are part of the unstructured description in the physician's personal profile. Therefore, only the four topics that are part of the unstructured description in the physician's personal profile will be further described.

**Table 2:** Most relevant words related to topics in the physicians' personal profiles.

**Topics**	**Top 15 words in each topic (in English)**	**Top 15 words in each topic (in Chinese)**
Academic rank and Clinic title	Graduate, professional, chief physician, professor, work, associate chief physician, master, graduate student, advisor, director, engaged, PhD, attending physician, learn, associate professor	毕业, 专业, 主任医师, 教授, 工作, 副主任医师, 硕士, 研究生, 导师, 主任, 从事, 博士, 主治医师, 进修, 副教授
Research ability	Publish, article, award, project, participation, host, SCI, project, technology, fund, access, journal, National Natural Science Foundation, research, core	发表, 论文, 等奖, 课题, 参与, 主持, SCI, 项目, 科技, 基金, 获得, 期刊, 国家自然科学基金, 科研, 核心
Foreign experience	Research, study, America, center, international, participation, technology, learn, visiting scholar, conduct, training, influence, Japan, Germany, conference	研究, 学习, 美国, 中心, 国际, 参加, 技术, 进修, 访问学者, 进行, 培训, 影响, 日本, 德国, 大会
Committee position	Member, committee, branch, professional, association, society, medical association, chairman, expert, youth, school group, editorial board, standing committee, member, standing committee	委员, 委员会, 分会, 专业, 协会, 学会, 医学会, 主任委员, 专家, 青年, 学组, 编委, 常委, 会员, 常务委员
Clinical experience	Be expert in, work, rich, engaged, technology, experience, clinical experience, development, patient, long- term, proficiency, first, special, more than 10 (or twenty) years, many years	擅长, 工作, 丰富, 从事, 技术, 经验, 临床经验, 开展, 患者, 长期, 熟练掌握, 率先, 特别, 余年, 多年

### Applications in Information Disclosure

We conducted regression analysis of our sample data according to our proposed model, and the results are shown in Table 3. We report the standardized regression coefficients, standard errors, and significant levels for all variables. First, we examine factors that affect user/patient ratings (HOT) that are under the control of the physicians' divisions. As indicated by the corresponding outcomes shown in the column for Model 0a, the length of the physician's profile (WORD) and the physician's online contribution (CONTR) have a significant and positive impact on user/patient ratings (HOT), with coefficients of 0.052 (*p* < 0.001) and 0.060 (*p* < 0.001), respectively. However, the physician's tenure with Haodf (TIME) shows a significantly negative impact on user/patient ratings (HOT) (β = −0.010, *p* < 0.001). In addition, we also find positive effects of the physician's clinic title (CT) (β = 0.051, *p* < 0.001), academic rank (AT) (β = 0.061, *p* < 0.001), and hospital level (HL) (β = 0.148, *p* < 0.001) on user/patient ratings (HOT). The *R*-Squared of Model 0a is 33.0%; that is, the model is able to explain a substantial amount of the variance in the dependent variable (i.e., HOT). Second, we further examine factors that affect page views (VIEW) under the same control of other variables. The relevant results are shown in the column for Model 0b. The coefficients of length of the physician's profile (WORD), physician's online contribution (CONTR), and physician's tenure with Haodf (TIME) have significantly positive impacts on page views (VIEW). We also find that the effects of the coefficients of clinic title (CT) and academic rank (AT) are significantly positive, and the effect of the coefficient of hospital level (HL) is significant negative. The *R*-Squared of Model 0b is 88.3%, which means that these variables can effectively explain even more of the variation of the dependent variable (i.e., VIEW).

**Table 3:** Results of the basic regression model.

**Variables**	**Model 0a: HOT Coefficient (SE)**	**Model 0b: VIEW Coefficient (SE)**
Intercept	3.002^***^ (0.009)	6.154^***^ (0.025)
WORD	0.052^***^ (0.002)	0.102^***^ (0.005)
CONTR	0.060^***^ (0.001)	0.539^***^ (0.002)
TIME	−0.010^***^ (0.001)	0.234^***^ (0.002)
CT	0.051^***^ (0.004)	0.032^***^ (0.010)
AT	0.061^***^ (0.003)	0.076^***^ (0.008)
HL	0.148^***^ (0.004)	−0.074^***^ (0.009)
DIV: Surgery	−0.007 (0.005)	−0.099^***^ (0.013)
DIV: Gynecology and obstetrics	−0.005 (0.007)	0.158^***^ (0.019)
DIV: Pediatrics	0.067^***^ (0.006)	−0.027 (0.016)
DIV: Orthopedics	0.028^***^ (0.006)	−0.095^***^ (0.017)
DIV: Ophthalmology	0.032^***^ (0.008)	0.040 (0.021)
DIV: Oral health	0.162^***^ (0.008)	0.126^***^ (0.022)
DIV: Cancer	0.071^***^ (0.009)	−0.119^***^ (0.023)
DIV: Traditional Chinese medicine	0.204^***^ (0.006)	0.190^***^ (0.016)
DIV: Others	−0.012^*^ (0.005)	0.085^***^ (0.014)
Adjusted R-squared	0.330	0.833

*** *Significant at 0.1%;*

** significant at 1%;

* *significant at 5%.*

*SE denotes standard error*.

Table 4 presents the results of the four models, with the other variables being the same, focusing on the topic model variables. The results indicate that REAEARCH and FEXP had a significant positive impact on HOT, with coefficients of 0.074 (*p* < 0.001) and 0.090 (*p* < 0.001), respectively. However, CEXP showed a significant negative impact on HOT (β = −0.014, *p* < 0.001). Finally, COMM had no significant impact on HOT.

**Table 4:** Results for the topic model applied to HOT.

**Variables**	**Model *1*a Coefficient (SE)**	**Model *2*aCoefficient (SE)**	**Model *3*a Coefficient (SE)**	**Model *4*a Coefficient (SE)**
RESEARCH	0.074^***^ (0.004)			
FEXP		0.090^***^ (0.004)		
COMM			−0.005 (0.004)	
CEXP				−0.014^***^ (0.003)
Intercept	3.050^***^ (0.009)	3.072^***^ (0.010)	3.000^***^ (0.009)	2.999^***^ (0.009)
WORD	0.039^***^ (0.002)	0.036^***^ (0.002)	0.052^***^ (0.002)	0.053^***^ (0.002)
CONTR	0.060^***^ (0.001)	0.059^***^ (0.001)	0.060^***^ (0.001)	0.060^***^ (0.001)
TIME	−0.010^***^ (0.001)	−0.011^***^ (0.001)	−0.010^***^ (0.001)	−0.010^***^ (0.001)
CT	0.054^***^ (0.004)	0.051^***^ (0.004)	0.051^***^ (0.004)	0.053^***^ (0.004)
AT	0.057^***^ (0.003)	0.057^***^ (0.003)	0.062^***^ (0.003)	0.061^***^ (0.003)
HL	0.144^***^ (0.004)	0.144^***^ (0.004)	0.148^***^ (0.004)	0.148^***^ (0.004)
DIV: Surgery	−0.008 (0.005)	−0.011^*^ (0.005)	−0.007 (0.005)	−0.007 (0.005)
DIV: Gynecology and obstetrics	0.001 (0.007)	0.002 (0.007)	−0.005 (0.007)	−0.004 (0.007)
DIV: Pediatrics	0.073^***^ (0.006)	0.071^***^ (0.006)	0.067^***^ (0.006)	0.068^***^ (0.006)
DIV: Orthopedics	0.027^***^ (0.006)	0.018^**^ (0.006)	0.028^***^ (0.006)	0.027^***^ (0.006)
DIV: Ophthalmology	0.033^***^ (0.008)	0.025^**^ (0.008)	0.031^***^ (0.008)	0.032^***^ (0.008)
DIV: Oral health	0.163^***^ (0.008)	0.155^***^ (0.008)	0.162^***^ (0.008)	0.161^***^ (0.008)
DIV: Cancer	0.067^***^ (0.009)	0.066^***^ (0.008)	0.071^***^ (0.009)	0.070^***^ (0.009)
DIV: Traditional Chinese medicine	0.213^***^ (0.006)	0.218^***^ (0.006)	0.204^***^ (0.006)	0.203^***^ (0.006)
DIV: Others	−0.009 (0.005)	−0.008 (0.005)	−0.012^*^ (0.005)	−0.012^*^ (0.005)
Adjusted R-squared	0.337	0.340	0.330	0.330

*** *Significant at 0.1%;*

** *significant at 1%;*

* *significant at 5%.*

*SE denotes standard error*.

Table 5 displays the results of the VIEW associated regression analysis under the same control of other variables. REAEARCH had a significant negative impact on VIEW (β = −0.055, *p* < 0.001). Conversely, FEXP and COMM had a significant positive impact on VIEW, with coefficients of 0.027 (*p* < 0.01) and 0.077 (*p* < 0.001), respectively. However, CEXP had no significant impact on VIEW.

**Table 5:** Results for the topic model applied to VIEW.

**Variables**	**Model 1b Coefficient**	**Model 2b Coefficient**	**Model 3b Coefficient**	**Model 4b Coefficient**
	**(SE)**	**(SE)**	**(SE)**	**(SE)**
RESEARCH	−0.055^***^ (0.010)			
FEXP		0.027^**^ (0.010)		
COMM			0.077^***^ (0.011)	
CEXP				−0.014 (0.008)
Intercept	6.119^***^ (0.026)	6.175^***^ (0.026)	6.196^***^ (0.025)	6.151^***^ (0.025)
WORD	0.111^***^ (0.005)	0.097^***^ (0.005)	0.093^***^ (0.005)	0.103^***^ (0.005)
CONTR	0.539^***^ (0.002)	0.539^***^ (0.002)	0.539^***^ (0.002)	0.539^***^ (0.002)
TIME	0.234^***^ (0.002)	0.234^***^ (0.002)	0.234^***^ (0.002)	0.234^***^ (0.010)
CT	0.030^***^ (0.010)	0.032^**^ (0.010)	0.026^**^ (0.010)	0.034^***^ (0.010)
AT	0.079^***^ (0.008)	0.074^***^ (0.008)	0.070^***^ (0.008)	0.075^***^ (0.008)
HL	−0.071^***^ (0.009)	−0.076^***^ (0.009)	−0.074^***^ (0.009)	−0.075^***^ (0.009)
DIV: Surgery	−0.099^***^ (0.013)	−0.100^***^ (0.013)	−0.096^***^ (0.013)	−0.100^***^ (0.013)
DIV: Gynecology and obstetrics	0.153^***^ (0.019)	−0.160^***^ (0.019)	0.161^***^ (0.019)	0.159^***^ (0.019)
DIV: Pediatrics	−0.032^*^ (0.016)	−0.026 (0.016)	−0.026 (0.016)	−0.027 (0.016)
DIV: Orthopedics	−0.094^***^ (0.017)	−0.098^***^ (0.017)	−0.092^***^ (0.017)	−0.096^***^ (0.017)
DIV: Ophthalmology	0.039 (0.021)	0.038 (0.021)	0.047^*^ (0.021)	0.041 (0.021)
DIV: Oral health	0.125^***^ (0.022)	0.124^***^ (0.022)	0.128^***^ (0.022)	0.123^***^ (0.022)
DIV: Cancer	−0.116^***^ (0.023)	−0.120^***^ (0.023)	−0.120^***^ (0.023)	−0.120^***^ (0.023)
DIV: Traditional Chinese medicine	0.183^***^ (0.016)	0.194^***^ (0.016)	0.186^***^ (0.016)	0.190^***^ (0.016)
DIV: Others	0.083^***^ (0.014)	0.086^***^ (0.014)	0.086^***^ (0.014)	0.085^***^ (0.014)
Adjusted R-squared	0.833	0.833	0.833	0.833

*** *Significant at 0.1%;*

** *significant at 1%;*

* *significant at 5%*.

*SE denotes standard error*.

## Discussions

### Theoretical Implications

This study is the first to use the LDA approach to extract latent dimensions from physicians' profile-generated data. It provides several theoretical contributions to the literature. First, we found that the introductions provided by physicians in the OHC allowed for the extraction of five primary topics, namely “academic rank and Clinic title,” “research ability,” “foreign experience,” “committee position,” and “clinical experience.” Other than the first topic (the physician's academic rank and clinic title), the topics are unstructured descriptions in the physician's profile. These findings advance our knowledge of information quality and have practical implications for purveyors of the OHC.

Second, the quality of the physician is very important to both purveyors and patients. We use the ratings to assess previous users' satisfaction with the quality of the physician (Li and Hitt, 2008). We conduct a regression analysis to test our proposed model. The results show that physicians' mentioning “research ability” and “foreign experience” was significantly positively correlated with the ratings. Overall, our findings suggest that physicians' “research ability” and “foreign experience” are signals of the quality of physicians to patients. The higher the quality of the physician, the higher the levels of patient satisfaction. These results are similar to those of recent meta-analysis studies (Blut et al., 2015). Surprisingly, physicians' mentioning “clinical experience” has a significant negative correlation with the ratings. This negative effect may come from the disconfirmation of belief, which is the difference between perceived performance and expectations (Richins and Bloch, 1991; Foumier and Mick, 1999). Intuitively, higher expectation or lower perceived performance induces greater disconfirmation of belief. According to Expectation-Confirmation Theory (ECT; Oliver, 1980), patients often collect and evaluate physicians' information from their profiles before making a decision, and then they form their own expectations. When a patient receives information that a physician has more clinical experience, he/she has a higher expectation for the physician, which may lead to negative disconfirmation of belief. Therefore, when a physician mentions that he/she has rich clinical experience, there is a significant negative impact on the patient's satisfaction.

Finally, from the perspective of the purveyors (i.e., Haodf), physicians' attracting more page views can create higher firm values (Demers and Lev, 2001; Dewan et al., 2002; Luo et al., 2013). Thus, we further explored the factors that affect page views. We found that physicians' mentioning “foreign experience” and “committee position” has a significant positive correlation with page views. However, “research ability” has a significant negative correlation with page views. Generally speaking, when a physician mentions that he/she has published an SCI article, or has received project support, the patient may not understand that this implies the physician's hard work and professional performance. If the physician's profile uses too many technical terms, the patient will not understand them and will not be attracted to browse. This may be the cause of the significant negative correlation between research ability and page views.

### Managerial Implications

The study has several valuable implications for management practices. First, for website managers, this study has analyzed unstructured data to extract physician information, a technique which can provide practitioners with information about website management and design strategies. For example, extracted topics can be utilized in addition to structural data. In addition, we found that academic achievement has a negative impact on page views, which may result from patients not understanding physicians' academic achievement, implying that website managers might consider explaining these terms in more detail.

Second, for physicians or hospitals, the dimensions of physician's introductions can be taken as a basis for determining consumer satisfaction, physician page views, and ad content design. In our context, exploring what kind of physician's image can bring greater satisfaction or attract more patients, provide website hosting or hospital managers understand how to properly improve the image of physicians.

Finally, for marketers in general, although this study was conducted in the context of OHC, the LDA can be used to analyze the unstructured information provided about other products. By extracting useful information from unstructured data, more accurate product positioning and appropriate marketing strategies can be developed to help companies win against the competition.

### Limitations and Future Research Directions

There are some limitations to this study as well as indications of possible directions for future research. First, all the empirical data were collected from www.haodf.com. This website is a representative OHC in China, which means that our findings may reflect only the Chinese OHC context. Past research indicates that culture is an important key factor affecting consumer behavior (De Mooij, 2010; De Mooij and Hofstede, 2010). Therefore, future study should be conducted with more diverse samples to improve the generalizability of the research results (Tang, 2017) and to make possible a comprehensive understanding of the marketing communication mix in a cross-cultural setting. Second, this study focuses only on physicians' profiles in the OHC, but it could be extended to other products (i.e., books, CDs, and DVDs). Future research can obtain unstructured data related to other products from news reports, advertisement copy, and other textual documents to extract useful information. Finally, different types of social media may affect the nature of interactions and influence consumers' perceptions and beliefs about advertising (Prendergast et al., 2009). Johnston et al. (2018) provide an insight into the potential of social media types to moderate the effect of belief on attitude and value. A possible extension of this work would be to investigate across products to shed light on which products are most affected by which communication channels (e.g., online forums of products, blogs, social media, email, and online catalogs); this would help businesses to efficiently allocate their resources.

## Conclusion

Understanding strike of the factors that influence physician ratings and page views is important for the continued growth of online healthcare communities. This study used the LDA model to obtain five latent physician characteristics from a large number of physician profiles collected, i.e., physician's academic rank and clinic title, research ability, foreign experience, committee position, and clinical experience. Except for the first one, which is a frequently used characteristic in past OHC studies, others were less frequently mentioned. Through regression analysis, we found that physicians' mention of their research ability and foreign experience had a significant positive effect on physician ratings but mentioning of clinical experience had a significant negative effect on physician ratings. In addition, physician mentions of foreign experience and committee position had a significant positive impact on page views, but physician mentions of research ability had a significant negative impact on page views. For OHC managers, these findings could be incorporated into the recommended system to improve physician ratings and page views. Overall, this study provides a new perspective on OHC-related research, in that text mining can be used to extract new features from physician profiles for further analysis.

## Data Availability Statement

The original contributions presented in the study are included in the article/supplementary material, further inquiries can be directed to the corresponding author.

## Author Contributions

XW: writing—reviewing and editing and conceptualization. Y-TH: investigation, resources, data curation, methodology, formal analysis, investigation, visualization, and writing—original draft preparation. Both authors contributed to the article and approved the submitted version.

## Funding

This work was supported by Ministry of Education Project of Humanities and Social Sciences (No. 20YJCZH199).

## Conflict of Interest

The authors declare that the research was conducted in the absence of any commercial or financial relationships that could be construed as a potential conflict of interest.

## Publisher's Note

All claims expressed in this article are solely those of the authors and do not necessarily represent those of their affiliated organizations, or those of the publisher, the editors and the reviewers. Any product that may be evaluated in this article, or claim that may be made by its manufacturer, is not guaranteed or endorsed by the publisher.

## Footnotes

^1^The most common definition of eWOM is “any positive or negative statement made by potential, actual, or former customers about a product or company, which is made available to a multitude of people and institutions via the Internet” (Hennig-Thurau et al., 2004).

^2^The Haodf website (hao dai fu means “good doctor” in Chinese) was the earliest online physician review website and has been in operation in China since 2006. As of October 2021, the Haodf website contains information of 860,000 doctors from 9,780 hospitals. Among them, 240,000 doctors are registered in their real names on the platform and has served more than 74 million patients in total. Users can conveniently reach doctors through multiple platforms such as APP, PC version website, mobile version website and WeChat mini-program to solve various medical problems in the form of online service and offline consultation.
